# Spinal cord compression is associated with brain plasticity in degenerative cervical myelopathy

**DOI:** 10.1093/braincomms/fcab131

**Published:** 2021-06-22

**Authors:** Alicia E Cronin, Sarah A Detombe, Camille A Duggal, Neil Duggal, Robert Bartha

**Affiliations:** 1 Department of Medical Biophysics, The University of Western Ontario, London, Ontario N6A 3K7, Canada; 2 Centre for Functional and Metabolic Mapping, Robarts Research Institute, The University of Western Ontario, London, Ontario N6A 3K7, Canada; 3 Department of Clinical Neurological Sciences, University Hospital, London Health Sciences Centre, London, Ontario N6A 5A5, Canada

**Keywords:** cervical myelopathy, cortical plasticity, motor cortex, functional MRI, conventional T_2_-weighted MRI

## Abstract

The impact of spinal cord compression severity on brain plasticity and prognostic determinates is not yet fully understood. We investigated the association between the severity of spinal cord compression in patients with degenerative cervical myelopathy, a progressive disease of the spine, and functional plasticity in the motor cortex and subcortical areas using functional magnetic resonance imaging. A 3.0 T MRI scanner was used to acquire functional images of the brain in 23 degenerative cervical myelopathy patients. Patients were instructed to perform a structured finger-tapping task to activate the motor cortex to assess the extent of cortical activation. T_2_-weighted images of the brain and spine were also acquired to quantify the severity of spinal cord compression. The observed blood oxygen level-dependent signal increase in the contralateral primary motor cortex was associated with spinal cord compression severity when patients tapped with their left hand (*r* = 0.49, *P* = 0.02) and right hand (*r* = 0.56, *P* = 0.005). The volume of activation in the contralateral primary motor cortex also increased with spinal cord compression severity when patients tapped with their left hand (*r* = 0.55, *P* = 0.006) and right hand (*r* = 0.45, *P* = 0.03). The subcortical areas (cerebellum, putamen, caudate and thalamus) also demonstrated a significant relationship with compression severity. It was concluded that degenerative cervical myelopathy patients with severe spinal cord compression recruit larger regions of the motor cortex to perform finger-tapping tasks, which suggests that this adaptation is a compensatory response to neurological injury and tissue damage in the spinal cord.

## Teaser

Using T_2_-weighted spine MRI and brain fMRI, Cronin et al. reveal that patients with severe spinal cord compression recruit larger regions of the motor cortex to perform finger-tapping tasks, which could indicate that the observed cortical reorganization is a compensatory response to tissue damage in the spinal cord.

## Graphical Abstract

**Graphical Abstract:**
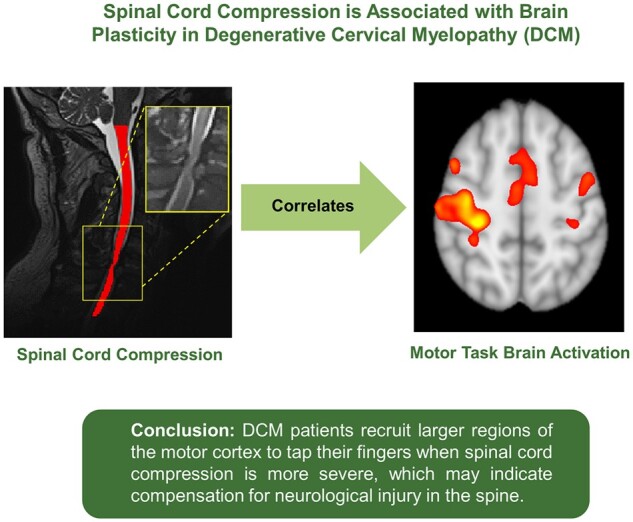

## Introduction

Cortical reorganization, in response to brain or spinal cord injury, may influence functional recovery and provide a compensatory mechanism to minimize functional deficits.^1^ Many functional magnetic resonance imaging (fMRI) studies have demonstrated that there is increased cortical activity in patients with spinal cord injuries in response to hand movement tasks.^1–5^ Studies in patients with spinal cord injuries have also found increased levels of activation in subcortical areas compared to controls.^2^^,^^6^^,^^7^ However, it is unclear if the plasticity occurring in these patients is associated with the severity of spinal cord compression and if severity of spinal cord compression influences functional recovery.

Degenerative cervical myelopathy (DCM) is one of the most common forms of spinal cord dysfunction, with the incidence and prevalence in North America estimated to be 41 and 605 per million, respectively.^8^ It is a unique model of spinal cord injury that becomes increasingly prevalent with age,^9^ can result in compression of the spinal cord,^10^ and can lead to neurological dysfunction.^11^ Surgical intervention, in the form of decompression surgery, is universally accepted as a preferred treatment option^12^ in patients with moderate to severe DCM.^13^^,^^14^ In many patients, surgical intervention can effectively prevent progression of neurological decline and improve functional outcome.^14^^,^^15^ In fact, approximately two-thirds of patients demonstrate some neurological recovery (e.g. upper limb function, lower limb function, and sphincter recovery) post-surgery.^15^ Unfortunately, select patients do not improve following surgery and some can continue to deteriorate. Identifying patients that do not respond to surgical intervention is a major unmet clinical need.

Predicting functional recovery and surgical outcome based on patient demographic factors such as age,^16^^,^^17^ level(s) of compression^18^ or duration of symptoms^19^ has proven unreliable. MRI parameters in the spine, such as hyperintensity on T_2_-weighted images^20^ and hypointensity on T_1_-weighted images^21^ have proven equally unreliable in predicting response to surgery and functional recovery. Interestingly, some studies have demonstrated that plasticity can occur in the brain when tissue damage occurs within the ascending and descending spinal cord fibre tracts.^2^^,^^22^ This finding suggests that the severity of spinal cord compression could be a useful prognostic indicator. To improve the prognostic determinates of DCM, the relationship between localized compression in the spinal cord, neuronal damage, cortical reorganization, and functional performance before and after surgery must be better understood.

Most fMRI studies in DCM have made group level comparisons between DCM patients and healthy controls, which treats DCM patients as a homogenous group.^1^^,^^3–5^^,^^23^ The aim of the current study was to determine if cortical activity differences in individual DCM patients, measured by fMRI, were associated with the severity of spinal compression. Understanding the relationship between spinal compression and brain plasticity may help to develop an objective prognostic indicator of surgical response. The overall goal of the current study was to determine if brain activity variations in individual DCM patients, measured by fMRI, were associated with the severity of spinal cord compression and neurological dysfunction, measured by validated clinical outcomes scores. We hypothesized that cortical and subcortical reorganization would be greater in patients with more severe spinal cord compression, and that patients with severe compression would have more impaired neurological function despite increased fMRI measured cortical activity.

## Materials and methods

### Participants and clinical evaluation

This study was approved by the Western University Health Sciences Research Ethics Board. Informed consent was obtained from each patient prior to the start of the study. Twenty-five patients [14 men, mean age (±SD) 63 ± 13.1 years, 24 right-handed] with symptoms of DCM and no other neurological disorders were recruited from November 2018 to February 2020 and participated in a 3.0 T MRI session before decompression surgery. All patients completed the validated measure for assessing disability resulting from myelopathy, called the modified Japanese Orthopaedic Association (mJOA) outcome measure.^24^ This metric measures the severity of clinical symptoms in patients with myelopathy by assessing motor dysfunction in the upper and lower extremities, bladder function, and sensory function in the upper extremities. Patients were graded on an 18-point scale, where upper motor function was scored out of 5, lower motor function was scored out of 7, upper sensory function was scored out of 3, and bladder function was scored out of 3.^24^ To be included in this study, DCM patients must have demonstrated some degree of hand dysfunction (4/5 or lower on upper mJOA score). Coincidently, all DCM patients also had varying degrees of gait dysfunction.

### Imaging protocol

Imaging was performed in DCM patients prior to their decompression surgery on a Siemens 3.0 T Prisma Fit MRI scanner using a 64-channel head and neck coil to acquire all data. Anatomical head images were acquired for each patient using a sagittal T_1_-weighted 3D magnetization-prepared rapid acquisition gradient echo sequence (9˚ flip angle, matrix size 256 × 256, number of slices = 175, 1 mm slice thickness, and repetition time/echo time 2300/2.98 ms). Blood oxygen level-dependent (BOLD) images were acquired using an interleaved echo planar imaging pulse sequence (720 × 720 acquisition matrix, 52 slices per volume, slice thickness 2.3 mm, repetition time/echo time 1000/30 ms, 40˚ flip angle). The total acquisition time of the BOLD scan was 5 min and 30 s for 330 volumes. Field maps were acquired to correct for signal distortions (slice thickness 3 mm, repetition time/echo time 500/4.92 ms, 60˚ flip angle). Finally, anatomical neck and spine images were also acquired for each patient using a T_2_-weighted sagittal 3D spin-echo sequence (slice thickness 0.9 mm, repetition time/echo time 2170/135 ms, flip angle 140˚, number of averages = 2).

### Study design

To activate the motor pathway, a block paradigm task, which included 11 segments (six resting and five active), was performed. All patients were instructed to perform a fingers-to-thumb pinch (duck quack) with their right hand in a button box. To control the frequency that patients were performing the task, visual cues were presented every 3 s during the 30 s task period. Compliance was ensured by the recording of the button presses using an in-house program created using MATLAB v. R2019b and Psychtoolbox v.3.0.15. This protocol was also repeated for the left hand.

### Imaging processing

Anatomical and functional images were preprocessed using the fMRI pipeline *fmriprep* version 1.4.1.^25^^,^^26^ Specifically, the anatomical T_1_-weighted head images were corrected for intensity non-uniformity using N4BiasFieldCorrection v2.1.0^27^ and skull-stripped using antsBrainExtraction.sh v2.1.0. Spatial normalization was performed through nonlinear registration with the antsRegistration tool of ANTS v2.1.0.^28^ The CSF, white-matter and gray-matter were all segmented on the brain extracted image to assist with registration. The functional images were corrected for motion using mcflirt [FMRIB Software Library (FSL) v5.0.9],^29^ slice timing corrections were applied using 3dTshift from AFNI v16.2.07,^30^ and field distortion corrections^31^ were performed. Co-registration to the corresponding T_1_-weighted image using boundary-based registration^32^ with 9 degrees of freedom was executed. The anatomical and functional data were all converted and reported in MNI space. For further details of the *fmriprep* pipeline, please refer to the online documentation: https://fmriprep.readthedocs.io/en/1.4.1/ (Accessed 23 July 2021).

To find brain activity related to our proposed block design, a general linear model of the whole brain was run separately for each of the patients. The data were spatially smoothed by convolving each slice with a 6 mm full-width-half maximum Gaussian kernel in FSL v6.03.^33^ We modelled the predictors of each patient by convolving the block paradigm boxcar function with a double-gamma hemodynamic response function and included the nuisance regressors to form the complete statistical model. The nuisance regressors were the motion-related parameters, consisting of three regressors for each translation direction and rotation direction. Cluster-based thresholding was performed (*Z* > 3.1, *P* = 0.001), where the *P*-value was corrected for multiple comparisons.^34^

**Figure 1:**
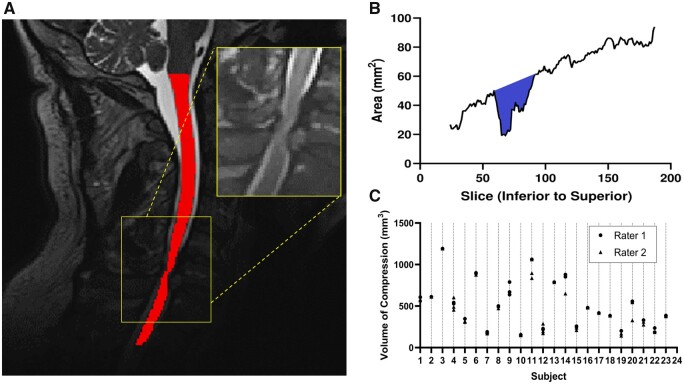
Volume of compression measurement. (**A**) T_2_-weighted image of the cervical spinal cord of a DCM patient showing the segmented cord in red, with the compression site displayed on the inset. (**B**) Line graph displaying the area of each axial slice of the segmented cord, from inferior to superior, with the coloured region the total compression volume measurement. (**C**) Dot plot of rater agreement, with an ICC of 0.977 for inter-rater reliability, first rater achieving an intra-rater reliability ICC of 0.996, and the second rater achieving an intra-rater reliability ICC of 0.967.

The cervical spinal cord was automatically segmented using the Spinal Cord Toolbox v4.2.2,^35^ specifically using the Deepseg^36^ module. This module is a deep-learning-based spinal cord segmentation module that uses two Convolutional Neural Networks, where the first detects the spinal cord centreline and the second performs the segmentation.^36^ Quality of the segmentation was manually checked on every axial slice using the FSL viewer, FSLeyes. An example of the segmented cord is demonstrated in Fig. 1A. Using the Spinal Cord Toolbox, the cross-sectional area was found for each axial slice of the spinal cord. Using custom MATLAB code, the total volume of the spinal cord in the compressed region was measured by identifying the limits of the compressed region using the rate of change of cord area, then summing the areas of each slice within the compressed region, as shown in Fig. 1B. To measure the reliability of the spinal cord volume measurement, two raters performed repeated measurements of cord compression using the approach described above. The first rater developed the metric (A.E.C.) and therefore had significant previous experience using the tool and the second rater had no previous experience in performing imaging measurements (C.A.D.). Each performed the measurement three separate times on the full dataset. For each measurement, raters were blinded, and the data were scrambled. The intraclass correlation (ICC) was computed to determine the intra and inter reliability.

### Statistical analysis

Brain regions of interest (ROIs) were selected based on previous studies using the same task block design and the demonstration of activation in these areas.^5^^,^^23^ The ROIs included were the cortical structures [primary motor cortex (M1), the primary somatosensory cortex (S1), the supplementary motor area (SMA) and the premotor cortex (PMC)] and the subcortical structures (cerebellum, putamen, caudate and thalamus). The contralateral region of each of the cortical ROIs and bilateral subcortical ROIs was chosen for each of the scans (right and left hand). Cortical ROIs were obtained from the probabilistic Harvard-Oxford cortical structural atlas and subcortical ROIs from the MNI Structural Atlas. The extent of activation in these regions was quantified using beta weights, which represent how much BOLD signal is associated to the task (% BOLD signal), and the volume of activation (VOA).

Pearson’s correlation coefficient (*r*) was used to test the hypothesis that the severity of spinal compression was correlated with neuronal activation characterized using % BOLD signal and VOA, and that the severity of spinal compression was correlated functionally using mJOA scores. Pearson’s correlation coefficient was also used to determine whether the duration of symptoms (Table 1) was correlated with neuronal activation (% BOLD signal and VOA).

**Table 1:** Demographic data and clinical characteristics of patients with DCM

Case	Age (mean 65 ± 13 years)	Sex	Site of impairment^a^		mJOA score (mean 9.9 ± 2.1)				
				Duration of symptoms (months)	Upper motor	Lower motor	Upper sensory	Bladder	Total score (max 18)
1	70	M	C3-4	7	2	2	1	1	6
2	51	M	C6-7	4	3	4	1	1	9
3	68	M	C5-6	15	3	4	1	3	11
4	57	F	C5-6	30	2	4	1	1	8
5	81	M	C5-6	12	2	4	1	3	10
6	77	M	C4-5	4	3	4	1	2	10
7	74	M	C4-5	24	4	6	1	3	14
8	60	M	C5-6	36	4	4	1	3	12
9	52	F	C3-4	12	1	6	0	1	8
10	82	F	C3-4	12	4	6	2	3	15
11	52	M	C5-6	4	4	3	1	2	10
12	70	M	C3-4	22	3	4	1	2	10
13	72	M	C3-4	24	2	4	1	2	9
14	77	M	C3-4	5	2	3	1	2	8
15	51	F	C4-5	11	4	4	1	2	11
16	34	F	C4-5	16	3	5	1	2	11
17	84	M	C3-4	7	2	3	1	2	8
18	79	F	C4-5	6	2	3	1	2	8
19	45	M	C3-4	5	3	4	1	2	10
20	72	F	C3-4	24	2	3	1	3	9
21	67	F	C4-5	3	2	4	1	2	9
22	56	F	C3-4	7	4	4	2	3	13
23	73	M	C3-4	3	3	3	1	2	9

mJOA, modified Japanese Orthopaedic Association.

a Location of compression where surgery was performed.

### Data availability

Data will be made available upon request, adhering to ethical guidelines.

## Results

The measurement of spinal cord volume was found to be highly reproducible. Specifically, the reliability of the spinal cord volume measurements between the two raters were characterized with an ICC of 0.977. Similarly, the intra-rater reliability of each rater was also substantial, with the first rater (A.E.C.) achieving an ICC of 0.996 and the second, less experienced, rater (C.A.D.) achieving an ICC of 0.967. Figure 1C provides the individual measurements for each subject to show the small variation observed.

**Figure 2:**
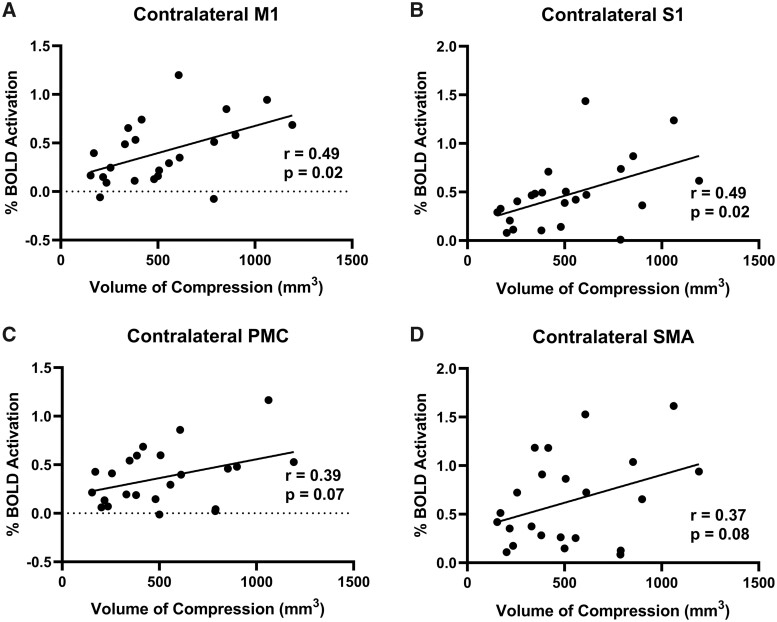
Left hand tapping BOLD signal and volume of compression. (**A**) The correlation between the % BOLD signal of the contralateral M1 and the spinal cord compression volume. (**B**) The correlation between the % BOLD signal of the contralateral S1 and the spinal cord compression volume. (**C**) The correlation between the % BOLD signal of the contralateral PMC and the spinal cord compression volume. (**D**) The correlation between the % BOLD signal of the contralateral SMA and the spinal cord compression volume.

**Figure 3:**
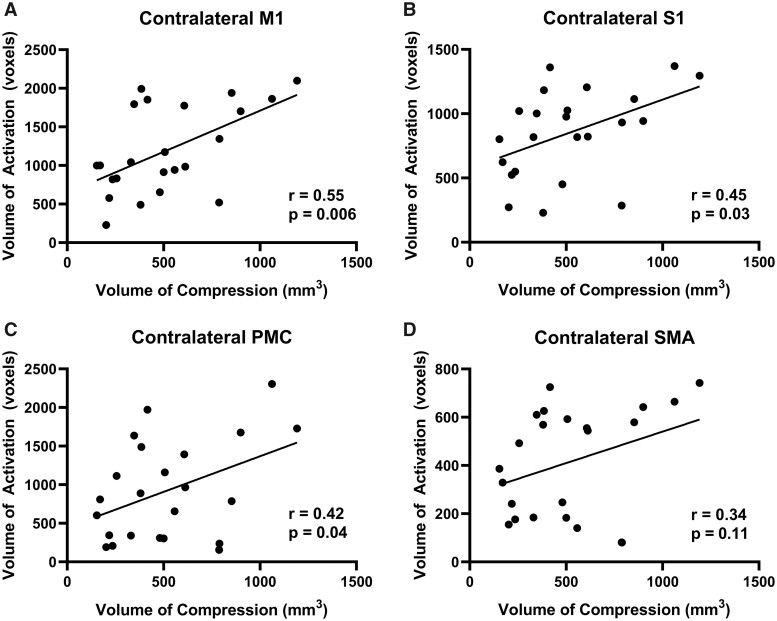
Left hand tapping volume of activation and volume of compression. (**A**) The correlation between the VOA of the contralateral M1 and the spinal cord compression volume. (**B**) The correlation between the VOA of the contralateral S1 and the spinal cord compression volume. (**C**) The correlation between the VOA of the contralateral PMC and the spinal cord compression volume. (**D**) The correlation between the VOA of the contralateral SMA and the spinal cord compression volume.

Two DCM patients were excluded from the study due to missing T_2_-weighted spine images and differing fMRI parameters. Demographics of this cohort of included patients are provided in Table 1. Supplementary Figure 1 highlights the differences in cortical activation patterns in individual participants with varying degrees of spinal cord compression. When DCM patients tapped with their left hand, motor network and subcortical activation was correlated with spinal cord compression volume. Specifically, in the contralateral M1, the % BOLD signal was significantly correlated with the total compression volume (*r* = 0.49, *P* = 0.02; Fig. 2A) and VOA was also significantly correlated with total compression volume (*r* = 0.55, *P* = 0.006; Fig. 3A). In the contralateral S1, the % BOLD signal was significantly correlated with the total compression volume (*r* = 0.49, *P* = 0.02; Fig. 2B) and VOA was also significantly correlated with total compression volume (*r* = 0.45, *P* = 0.03; Fig. 3B). In the associated motor areas (SMA and PMC), the total compression volume was only significantly correlated with the PMC VOA (*r* = 0.42, *P* = 0.04; Fig. 3C). Regarding subcortical brain areas, there was a significant correlation between spinal compression volume and % BOLD signal in the cerebellum (*r* = 0.56, *P* = 0.006; Supplementary Fig. 2A), the putamen (*r* = 0.57, *P* = 0.005; Supplementary Fig. 2B), the caudate (*r* = 0.67, *P* = 0.0004; Supplementary Fig. 2C) and the thalamus (*r* = 0.60, *P* = 0.003; Supplementary Fig. 2D). There was also a significant correlation between spinal compression volume and VOA in the cerebellum (*r* = 0.56, *P* = 0.006; Supplementary Fig. 3A), the putamen (*r* = 0.58, *P* = 0.004; Supplementary Fig. 3B), the caudate (*r* = 0.70, *P* = 0.0002; Supplementary Fig. 3C), and the thalamus (*r* = 0.63, *P* = 0.001; Supplementary Fig. 3D).

**Figure 4:**
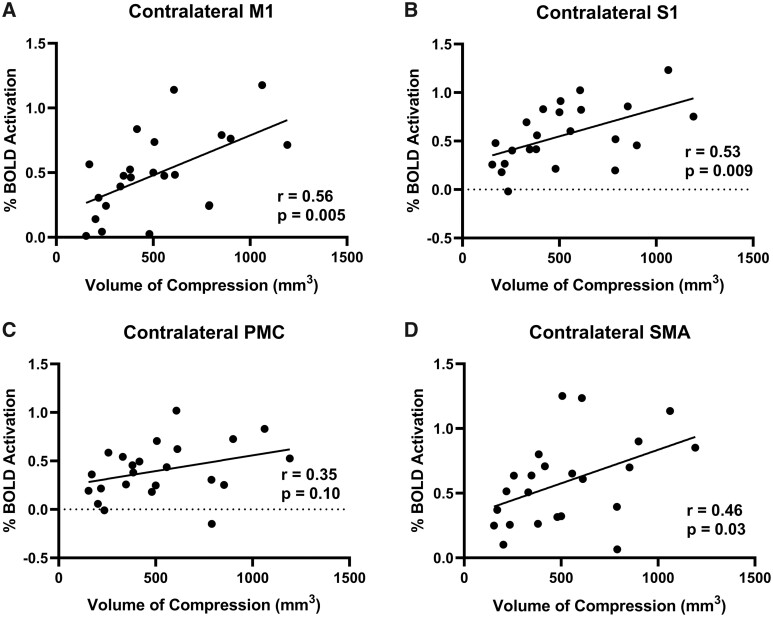
Right hand tapping BOLD signal and volume of compression. (**A**) The correlation between the % BOLD signal of the contralateral M1 and the spinal cord compression volume. (**B**) The correlation between the % BOLD signal of the contralateral S1 and the spinal cord compression volume. (**C**) The correlation between the % BOLD signal of the contralateral PMC and the spinal cord compression volume. (**D**) The correlation between the % BOLD signal of the contralateral SMA and the spinal cord compression volume.

**Figure 5:**
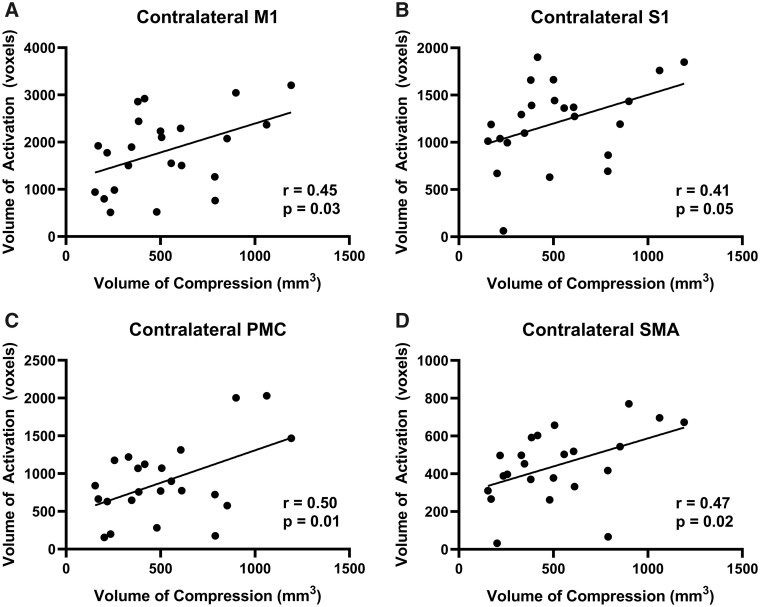
Right hand tapping volume of activation and volume of compression. (**A**) The correlation between the VOA of the contralateral M1 and the spinal cord compression volume. (**B**) The correlation between the VOA of the contralateral S1 and the spinal cord compression volume. (**C**) The correlation between the VOA of the contralateral PMC and the spinal cord compression volume. (**D**) The correlation between the VOA of the contralateral SMA and the spinal cord compression volume.

Similarly, when patients tapped with their right hand, cortical and subcortical activation was correlated with spinal cord compression volume. Specifically, in the contralateral M1 of the patients, a larger % BOLD signal was associated with a larger spine compression (*r* = 0.56, *P* = 0.005; Fig. 4A) and a larger VOA was also associated with a larger compression (*r* = 0.45, *P* = 0.03; Fig. 5A). In the contralateral S1, the % BOLD signal was significantly correlated with the total compression volume (*r* = 0.53, *P* = 0.009; Fig. 4B) and the VOA was close to significance with the total compression volume (*r* = 0.41, *P* = 0.05; Fig. 5B). In the contralateral PMC, the VOA was also significantly correlated with the total compression severity (*r* = 0.50, *P* = 0.01; Fig. 5C). Likewise, in the contralateral SMA, it was demonstrated that patients with a larger % BOLD signal also had a larger spine compression (*r* = 0.46, *P* = 0.03; Fig. 4D) and a larger VOA was also associated with a larger compression (*r* = 0.47, *P* = 0.02; Fig. 5D). Regarding the subcortical areas, a larger spinal cord compression volume was associated a higher % BOLD signal in the cerebellum (*r* = 0.50, *P* = 0.02; Supplementary Fig. 4A), the putamen (*r* = 0.70, *P* = 0.0002; Supplementary Fig. 4B), the caudate (*r* = 0.65, *P* = 0.0007; Supplementary Fig. 4C) and the thalamus (*r* = 0.52, *P* = 0.01; Supplementary Fig. 4D). Finally, total spinal cord compression volume was also significantly correlated with VOA in the cerebellum (*r* = 0.53, *P* = 0.01; Supplementary Fig. 5A), the putamen (*r* = 0.71, *P* = 0.0001; Supplementary Fig. 5B), the caudate (*r* = 0.73, *P* = 0.0001; Supplementary Fig. 5C) and the thalamus (*r* = 0.59, *P* = 0.003; Supplementary Fig. 5D).

**Figure 6:**
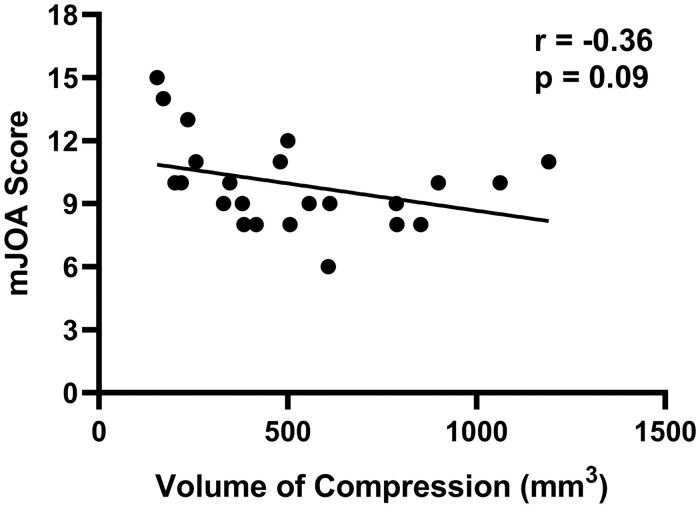
Correlation between mJOA score and volume of compression. The association between the neurological function in DCM patients measured by the mJOA score and spinal cord compression volume.

**Figure 7:**
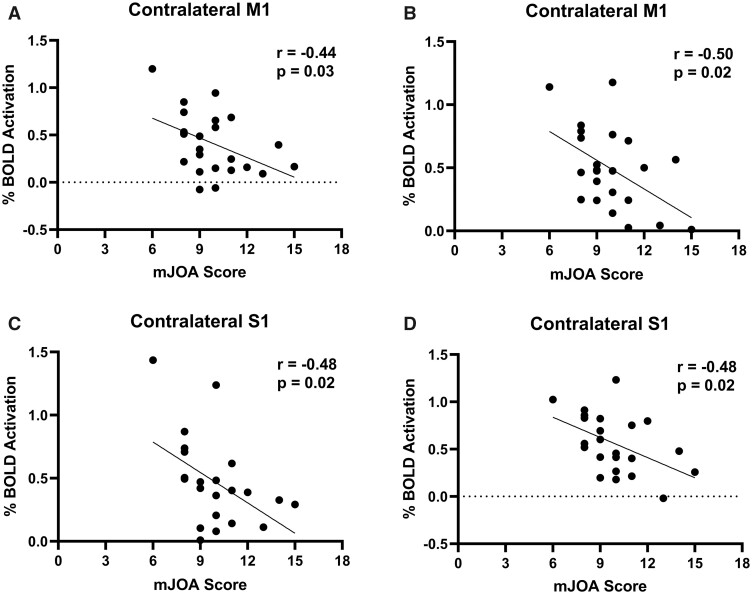
Correlation between % BOLD signal and mJOA. (**A**) The correlation between the % BOLD signal of the contralateral M1when tapping with the left hand and neurological function of the CSM patients measured by the mJOA score. (**B**) The correlation between the % BOLD signal of the contralateral M1 when tapping with the right hand and the mJOA score. (**C**) The correlation between the % BOLD signal of the contralateral S1 when tapping with the left hand and the mJOA score. (**D**) The correlation between the % BOLD signal of the contralateral S1 when tapping with the right hand and the mJOA score.

The mean mJOA score for the patient cohort was 9.9 ± 2.1 (mean ± SD). Analysis of compression severity and clinical scores (mJOA) did not demonstrate a significant relationship (*r* = −0.36, *P* = 0.09; Fig. 6). There was also no significant relationship between subcortical activation and clinical scores. However, analysis of the motor network and clinical scores (mJOA) demonstrated a significant relationship between activation and function. When patients tapped with their left hand, the contralateral M1 % BOLD signal was significantly correlated with the mJOA score (*r* = −0.44, *P* = 0.03; Fig. 7A), indicating that a higher function is associated with a smaller signal change. Likewise, in the contralateral S1, a smaller % BOLD signal was associated with a higher mJOA score (*r* = −0.48, *P* = 0.02; Fig. 7C). Furthermore, when patients were tapping with their right hand, the same significant relationships were demonstrated [contralateral M1 % BOLD signal (*r* = −0.50, *P* = 0.02; Fig. 7B), contralateral S1 % BOLD signal (*r* = −0.48, *P* = 0.02; Fig. 7D)]. There were no significant associations between any motor network activation and duration of symptoms.

## Discussion

In this study, conventional T_2_-weighted MRI was used to quantify spinal cord compression severity in DCM patients using a newly developed method with high reproducibility. The association between spinal cord compression and motor function was assessed using clinical scores. In addition, the association between spinal cord compression and activation of the motor network of the brain was assessed using fMRI in response to a finger tapping task. The results indicate that the total compression volume was positively correlated with the volume and magnitude of activation in several motor regions, including the M1, S1, PMC, SMA, cerebellum, putamen, caudate and thalamus. Additionally, mJOA scores were negatively correlated with the % BOLD signal in contralateral M1 and S1. To our knowledge, this is the first study in DCM that specifically explores the relationship between the severity of spinal cord compression and compensatory brain plasticity.

The current study demonstrates that DCM patients exhibit varying compensatory expansion of cortical activation *depending on the severity of spinal cord compression*. In many of the motor regions examined, there was a significant positive correlation between compression volume and activation levels, indicating that patients with greater spinal cord compression experience a larger compensatory expansion of activation or cortical recruitment. The expansion of the activated motor areas when DCM patients performed the controlled motor task may be related to rewiring of the axons of the lower limb extremities into the hand regions,^37^ which is driven by the use of the less affected part of the body to compensate for the difficulty with the instructed hand task. This effect has been shown in spinal cord injury patients, with an increase in handgrip related BOLD signal in the medial precentral gyrus, consistent with leg representation.^38^ The current study also demonstrated that patients with greater spinal cord compression have larger subcortical regions of activation. This increase in subcortical activation was previously suggested^6^ to be due to the reduction of afferent input from the spinal cord, which could lead to more complex processing of the remaining input, leading to greater activation. Since the subcortical regions examined are part of circuits that incorporate the cortical regions,^39^ activation pattern changes in subcortical regions could have a direct influence on the observed activation in the cortical regions.

There is mounting evidence from studies performing group-wise comparisons to control subjects or examining longitudinal changes in DCM subjects that DCM patients experience a reorganization of the motor areas in the brain. In one of the first studies demonstrating cortical reorganization in DCM patients, Holly et al.^1^ showed in four patients that there was an expansion of neuronal activity in the motor areas affected when performing either a wrist extension task or an ankle dorsiflexion task compared to healthy controls. Duggal et al.^3^ completed a study including 12 patients and 10 controls and performed both pre-operative and 6-month post-operative fMRI scans using a finger-tapping paradigm. It was found that patients demonstrated a larger VOA compared to controls in the precentral gyrus pre-operatively. Following surgery, this VOA difference between controls and DCM patients increased in this region. Hrabalek et al.^4^ completed a study involving seven patients and performed both pre-operative and 6-month post-operative fMRI scans using wrist flexions and extensions. It was found that there was significant activation in the dorsal M1, the adjacent secondary motor and sensory areas, and the cerebellum. Following surgery, there was a significant decrease in activation in the right parietal operculum and posterior temporal lobe.

In a larger study with 17 patients, Bhagavatula et al.^5^ also showed that compared to healthy controls, DCM patients had larger volumes of activation in their motor areas and cerebellum. Following decompression surgery, this cohort of patients demonstrated a decrease in activation compared to activation levels before surgery but remained higher than that of the control group. In a study that included 28 patients, Aleksanderek et al.^40^ demonstrated that there was a VOA difference pre-operatively between DCM patients with mild and moderate myelopathy, which was defined by mJOA. More specifically, the mild DCM patient group had a larger VOA in the postcentral gyrus compared to the moderate DCM group. This difference was no longer significant following surgery. Finally, Ryan et al.^23^ found that patients only exhibited a smaller VOA in the contralateral S1 compared to controls pre-operatively, which they attributed to the compression of the spinal cord attenuating signal transduction to the cortical motor networks. Overall, these studies provide evidence to support the notion that there is a change in cortical activity in the motor areas of DCM patients pre-operatively.

We have demonstrated that DCM patients with larger spinal compression volumes also have greater activation levels within the motor regions of the brain. The compression of the spine may induce pathophysiological changes in the spine that could impact recovery after surgery. For example, both primary mechanical and secondary biological injury in the spinal cord have been acknowledged to cause functional deficits in DCM. However, cellular changes within the spine have not been well defined during disease pathogenesis. It has been hypothesized that ischaemia and hypoxia, secondary to compression, are important pathophysiological mechanisms, however, direct in-vivo measurement of these conditions has been challenging in humans. Studies involving animal models of DCM^41^^,^^42^ and histological changes^43^^,^^44^ have provided indirect evidence of these pathophysiological mechanisms. However, the role of ischaemia and hypoxia in the spine in DCM disease progression and recovery is currently unknown.

The significant correlation between spinal compression volume and cortical plasticity is consistent with the presence of ischaemia and hypoxia in the spine. Previous studies have demonstrated that cortical reorganization can occur in the brain when there is injury within the ascending and descending fibre tracts within the spinal cord.^2^^,^^22^ Ischaemia and hypoxia can be caused by the disruption of vascular structures as a result of tissue compression. A study performed by Ellingson et al.^45^ found a decrease in blood flow in the region of the spinal cord that was compressed, supporting the hypothesis that spinal cord compression in DCM patients may result in ischaemia and hypoxia. Since greater compression likely induces greater ischaemia and hypoxia in the cord, it is reasonable to hypothesize that the observed cortical reorganization is a compensatory response to tissue damage in the spinal cord.

Our results also demonstrated that patients with a higher clinical score and functional ability, measured through mJOA, had lower activation in the brain motor areas. This result suggests that greater cortical recruitment may not necessarily translate into functional gain pre-surgery. This effect has also been demonstrated in subjects with spinal cord injuries, where subjects with better upper limb function showed lower levels of activation in the primary motor cortex region.^38^ This has also been shown in stroke patients, where patients with increased cortical activation in the sensorimotor cortex also demonstrated increased functional impairment.^46^ They attributed this finding to the clinical changes indirectly reflecting injury-induced adaptive cortical recruitment of undamaged motor control pathways.^46^

There are several limitations of the current study that are important to note. First, due to the high dimensionality and complexity of fMRI data, it is challenging to interpret single-subject results. One intermediate approach between group level analysis and individual analysis is to perform clustering of subgroups with similar activation characteristics. This method could be used to identify differences in DCM patients without the complexity of interpreting the single-subject data. In the future, this approach could be used to determine if one subgroup of patients demonstrates neurological recovery following decompression surgery. However, for this method to work effectively, a larger cohort of patients is needed. Second, this study included participants with compression sites ranging from C3-4 to C6-7. The site of compression may also account for some variance in the functional measures, and future studies with larger cohorts should examine this effect. Third, this in-vivo study was not designed to identify the extent and pathogenesis of cellular injury in the spinal cord. Future studies should be performed to directly quantify the extent of ischaemia and hypoxia in the cord and examine the relationship to tissue compression. Finally, it is currently unknown whether spinal cord compression measures, combined with measures of brain activation, could predict who will not respond favourably to spinal decompression surgery, but a longitudinal study should be performed to investigate.

## Conclusion

The current study indicates that DCM patients recruit larger regions of the motor cortex and subcortical areas to tap their fingers when spinal cord compression is more severe. This adaptation may compensate for neurological injury in the spine. Interestingly, the relationship between motor cortex activation patterns and function showed an inverse relationship indicating individuals with larger activation patterns had worse function. Taken together, these data suggest that individuals with more severe spinal cord compression exhibit larger brain activation patterns to complete motor tasks, but that this does not translate into improved function. Future studies should determine whether larger activation patterns confer an advantage for recovery following decompression surgery.

## Supplementary material

Supplementary material is available at *Brain Communications* online.

## Supplementary Material

fcab131_Supplementary_Data

## Abbreviations

BOLD =: blood oxygen level-dependent
DCM =: degenerative cervical myelopathy
fMRI =: functional magnetic resonance imaging
FSL =: FMRIB Software Library
ICC =: intraclass correlation
M1 =: primary motor cortex
mJOA =: modified Japanese Orthopaedic Association
PMC =: premotor cortex
ROI =: region of interest
S1 =: primary somatosensory cortex
SMA =: supplementary motor area
VOA =: volume of activation
