# Supplementary material for: Spinal cord compression is associated with brain plasticity in degenerative cervical myelopathy
Source: Brain Commun. 2021 Jun 22;3(3):fcab131. doi: 10.1093/braincomms/fcab131 (PMC8361426; doi:10.1093/braincomms/fcab131)
Supplement: fcab131_Supplementary_Data [file fcab131_supplementary_data.pdf]

Supplementary Information

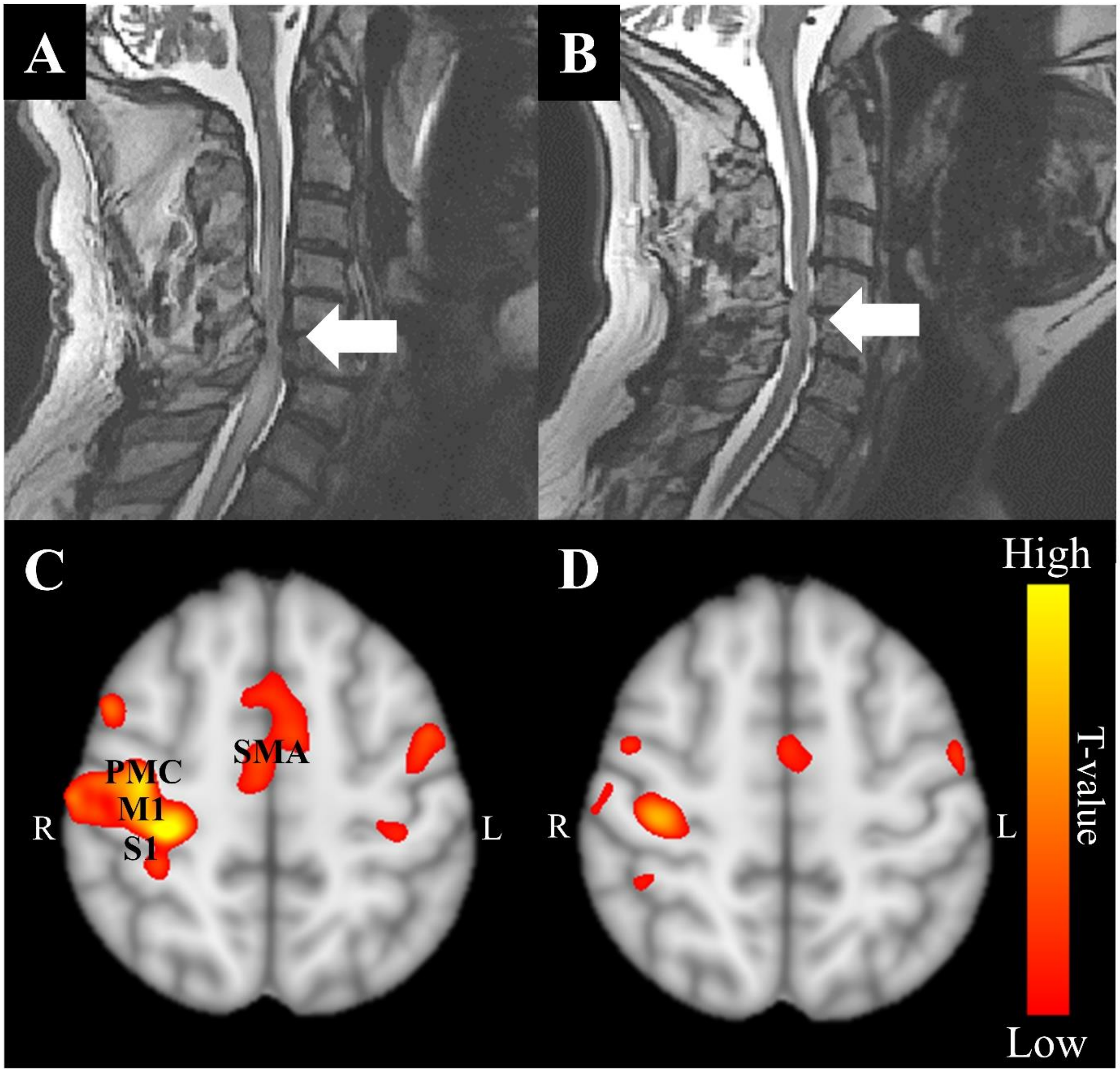

Supplementary Figure 1: fMRI activation map variations associated with spinal cord compression severity. **A:** Participant with a significant compression site (white arrow). **B:** A symptomatic participant with a lesser degree of spinal cord compression (white arrow). **C:** The corresponding fMRI activation map (left hand tapping) for the participant with severe compression. **D:** The corresponding fMRI activation map (left hand tapping) for the participant with less compression.

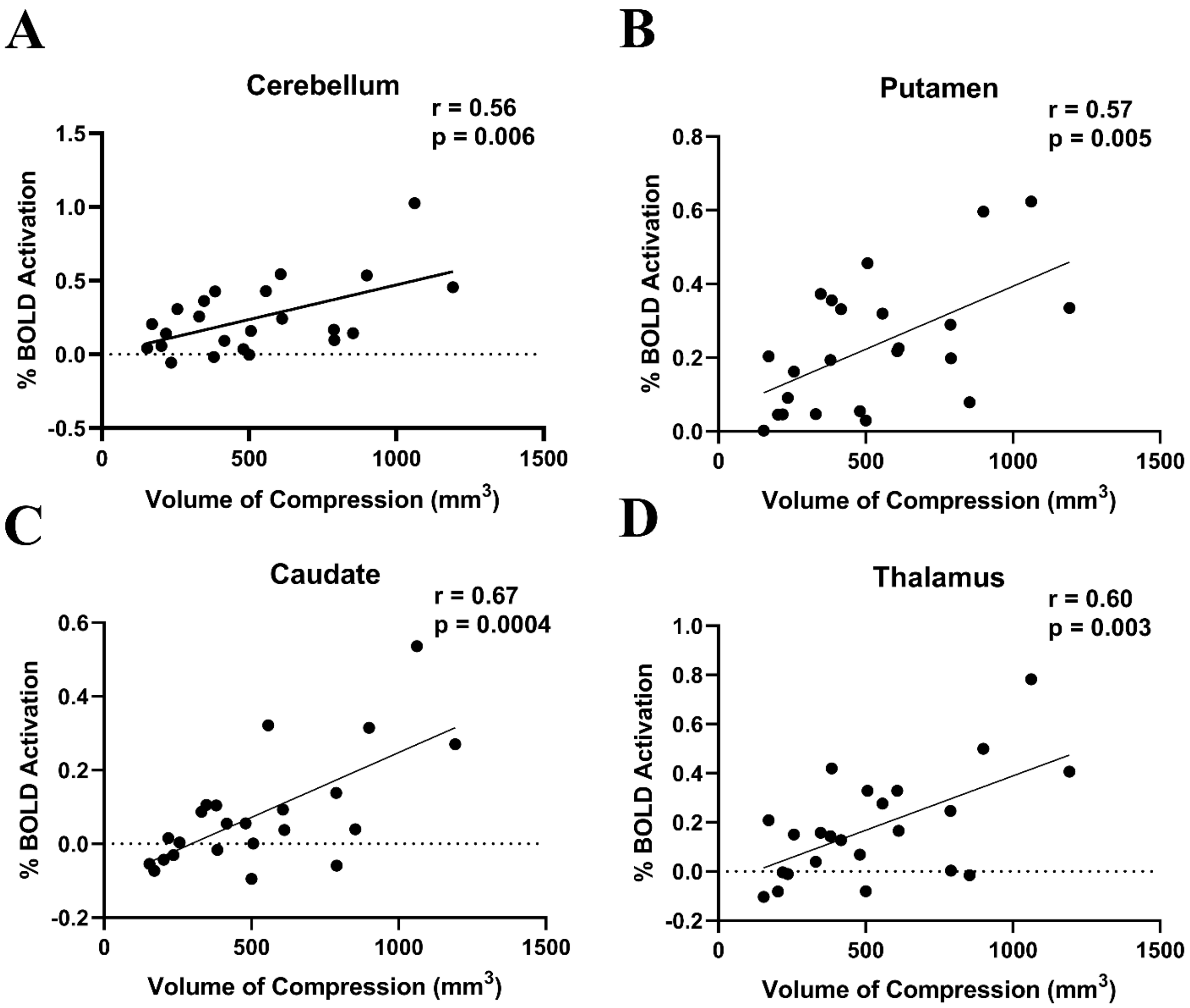

Supplementary Figure 2: Left hand tapping BOLD signal and volume of compression. A: The correlation between the % BOLD signal of the cerebellum and the spinal cord compression volume. **B:** The correlation between the % BOLD signal of putamen and the spinal cord compression volume. **C:** The correlation between the % BOLD signal of the caudate and the spinal cord compression volume. **D:** The correlation between the % BOLD signal of the thalamus and the spinal cord compression volume.

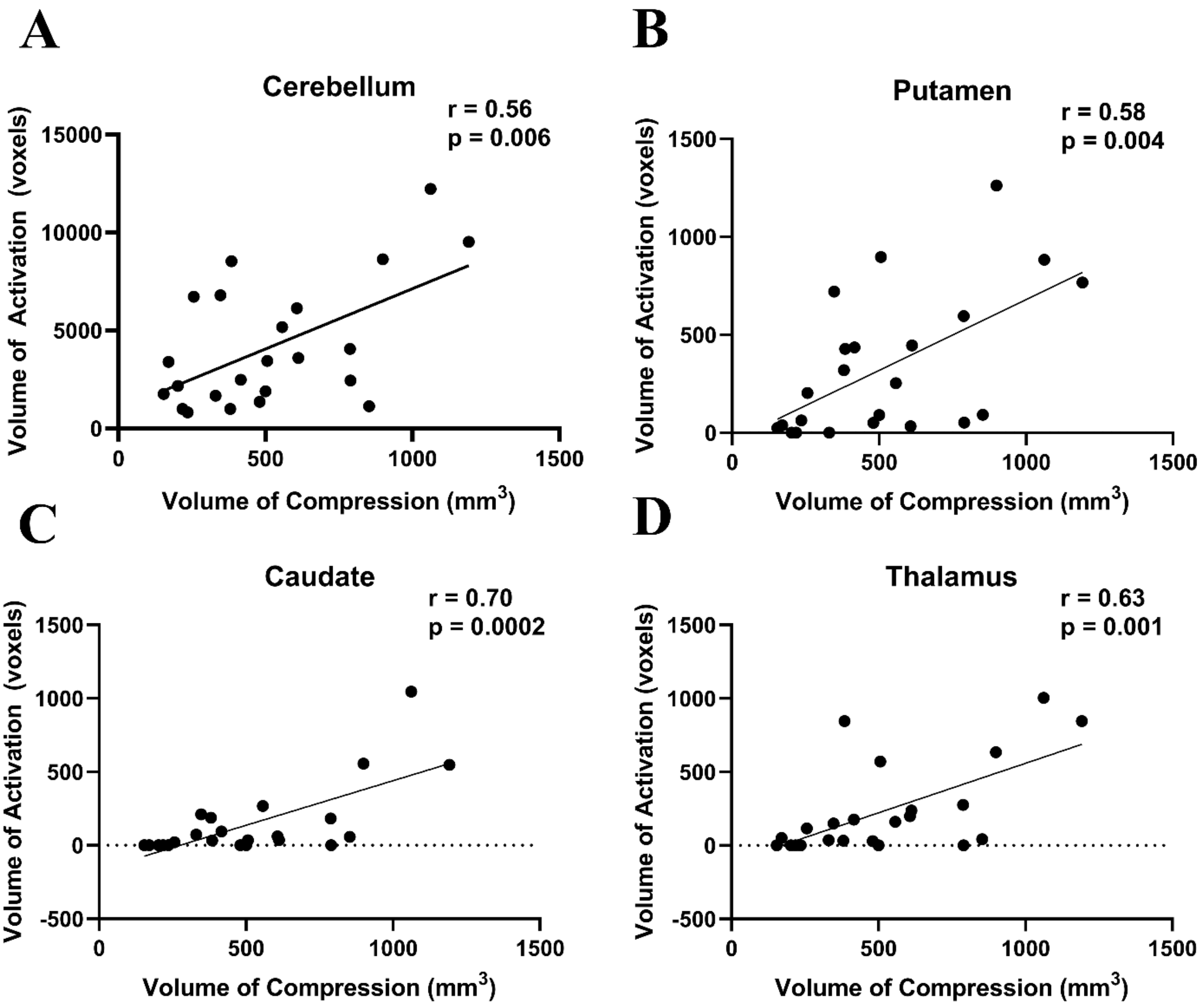

Supplementary Figure 3: Left hand tapping volume of activation (VOA) and volume of compression. A: The correlation between the VOA of the cerebellum and the spinal cord compression volume. **B:** The correlation between the VOA of putamen and the spinal cord compression volume. **C:** The correlation between the VOA of the caudate and the spinal cord compression volume. **D:** The correlation between the VOA of the thalamus and the spinal cord compression volume.

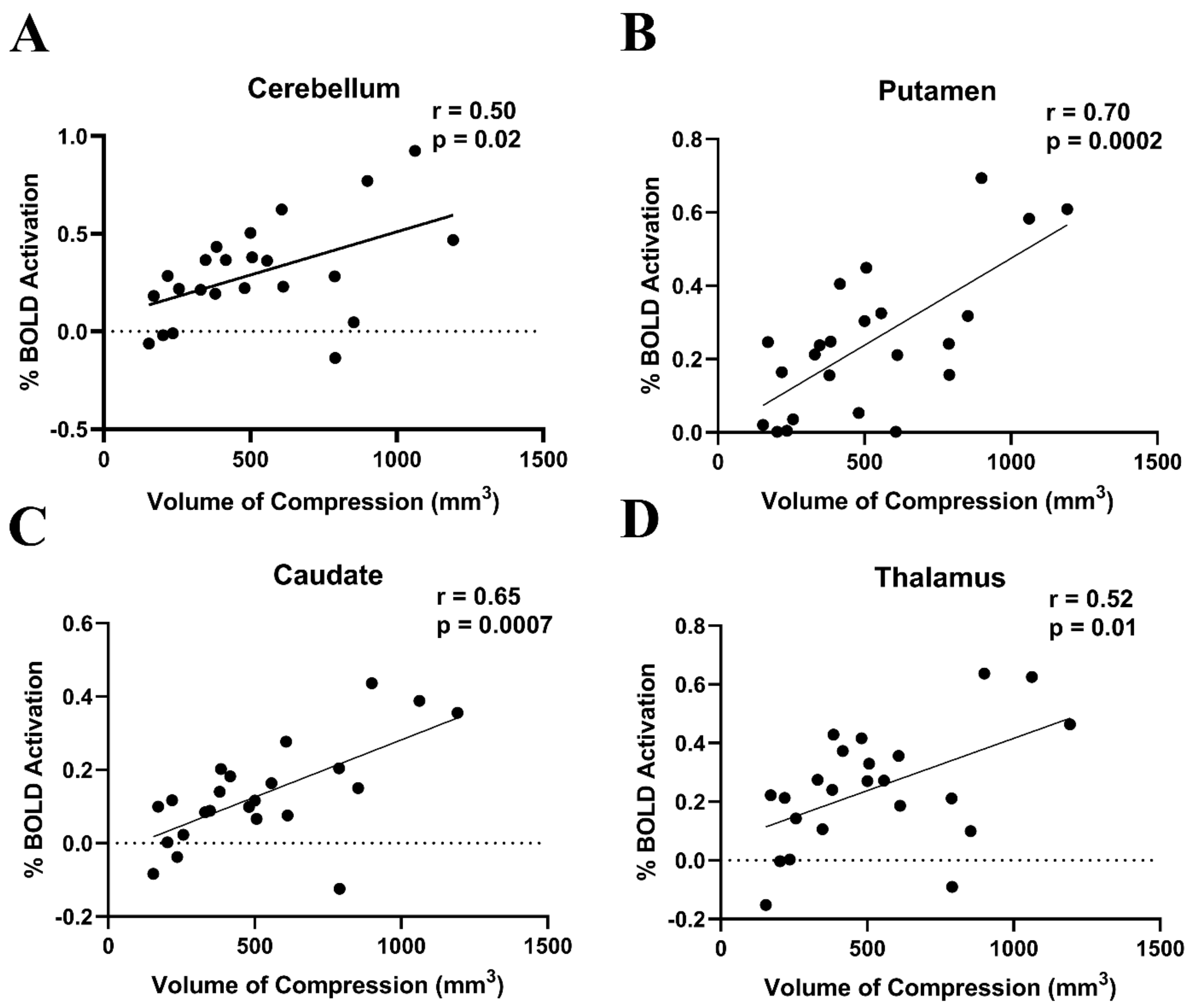

Supplementary Figure 4: Right hand tapping BOLD signal and volume of compression. A: The correlation between the % BOLD signal of the cerebellum and the spinal cord compression volume. **B:** The correlation between the % BOLD signal of putamen and the spinal cord compression volume. **C:** The correlation between the % BOLD signal of the caudate and the spinal cord compression volume. **D:** The correlation between the % BOLD signal of the thalamus and the spinal cord compression volume.

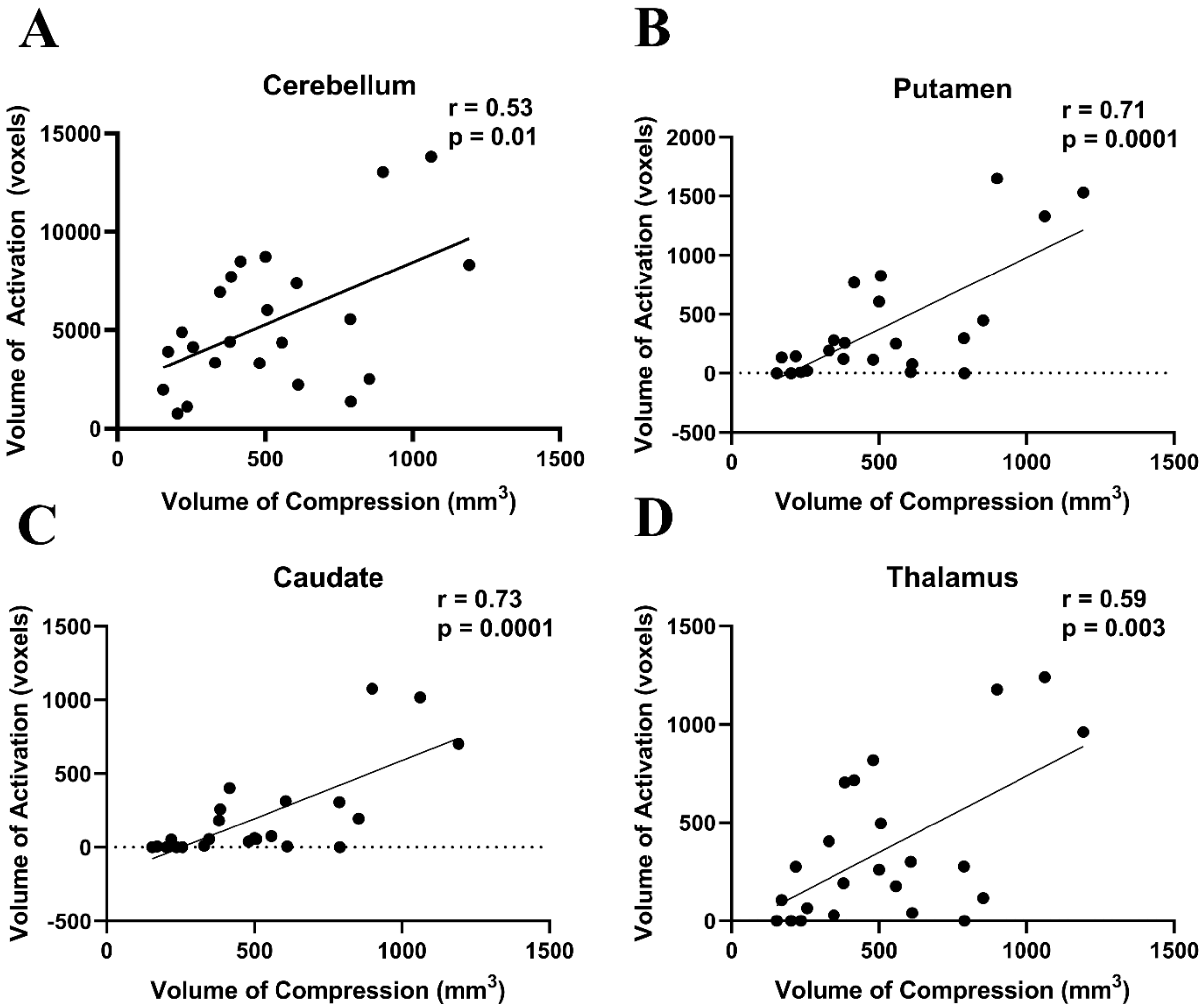

Supplementary Figure 5: Right hand tapping volume of activation (VOA) and volume of compression. A: The correlation between the VOA of the cerebellum and the spinal cord compression volume. **B:** The correlation between the VOA of putamen and the spinal cord compression volume. **C:** The correlation between the VOA of the caudate and the spinal cord compression volume. **D:** The correlation between the VOA of the thalamus and the spinal cord compression volume.

Supplementary Table 1: The measurements of the spinal cord compression volume (mm^3) found for each rater

Subject	Rater 1			Rater 2		
	Measure 1	Measure 2	Measure 3	Measure 1	Measure 2	Measure 3
1	607.3	607.3	607.3	582.0	626.7	569.9
2	611.8	611.8	611.8	611.8	611.8	611.8
3	1191.8	1191.8	1191.8	1191.8	1191.8	1191.8
4	542.2	529.4	529.4	604.2	491.0	455.4
5	346.8	346.8	346.8	317.9	314.9	308.0
6	899.3	892.4	892.4	876.4	875.4	905.7
7	170.7	189.2	189.2	169.8	169.8	189.2
8	500.5	500.5	500.5	500.5	499.7	472.6
9	789.4	669.0	638.7	662.2	660.9	660.9
10	153.9	153.9	153.9	148.6	162.9	153.9
11	1062.4	1062.4	1062.4	836.7	1062.4	896.8
12	217.9	228.1	228.1	173.2	196.5	288.1
13	788.2	788.2	788.2	788.2	788.2	788.2
14	852.6	852.6	879.8	651.0	651.0	879.8
15	256.7	256.7	256.7	239.1	256.7	210.4
16	480.6	480.6	486.6	480.6	486.6	480.6
17	416.7	416.7	416.7	416.7	416.7	417.3
18	384.2	384.2	384.2	384.2	384.2	384.2
19	201.8	201.8	201.8	143.5	143.5	168.9
20	557.2	557.2	557.2	545.7	328.5	557.2
21	330.6	330.6	330.6	310.4	276.2	279.2
22	235.4	186.0	235.4	186.0	186.0	186.0
23	380.4	385.9	385.9	385.9	376.7	373.8
